# A Strained 9-1-1 System and Threats to Public Health

**DOI:** 10.1007/s10900-015-0142-x

**Published:** 2015-12-24

**Authors:** Carolyn C. Cannuscio, Andrea L. Davis, Amelia D. Kermis, Yasin Khan, Roxanne Dupuis, Jennifer A. Taylor

**Affiliations:** Department of Family Medicine and Community Health, Perelman School of Medicine, University of Pennsylvania, Anatomy Chemistry Building, Room 145, 3620 Hamilton Walk, Philadelphia, PA 19104 USA; Department of Environmental and Occupational Health, Dornsife School of Public Health, Drexel University, Philadelphia, PA USA; Center for Public Health Initiatives, University of Pennsylvania, Philadelphia, PA 19104 USA

**Keywords:** Emergency medical technicians, Firefighters, Emergencies, Emergency treatment, Safety

## Abstract

The goal of this study was to understand safety climate in the United States (U.S.) fire service, which responded to more than 31 million calls to the 9-1-1 emergency response system in 2013. The majority of those calls (68 %) were for medical assistance, while only 4 % of calls were fire-related, highlighting that the 9-1-1 system serves as a critical public health safety net. We conducted focus groups and interviews with 123 firefighters from 12 fire departments across the United States. Using an iterative analytic approach supported by NVivo 10 software, we developed consensus regarding key themes. Firefighters concurred that the 9-1-1 system is strained and increasingly called upon to deliver Emergency Medical Services (EMS) in the community. Much like the hospital emergency department, EMS frequently assists low-income and elderly populations who have few alternative sources of support. Firefighters highlighted the high volume of low-acuity calls that occupy much of their workload, divert resources from true emergencies, and lead to unwarranted occupational hazards like speeding to respond to non-serious calls. As a result, firefighters reported high occupational stress, low morale, and desensitization to community needs. Firefighters’ called for improvements to the 9-1-1 system—the backbone of emergency response in the U.S.—including better systems of triage, more targeted use of EMS resources, continuing education to align with job demands, and a strengthened social safety net to address the persistent needs of poor and elderly populations.

## Introduction

The emergency medical services (EMS) system in the United States (U.S.) is fragmented, with various private and public entities responsible for dispatching calls, acting as first responders, and providing transport for medical emergencies [1]. Fire departments represent the majority (89 %) of first responders to arrive on the scene of an accident or emergency in large metropolitan areas [1]. Indeed, in 2013, fire departments nationwide responded to 31,644,500 calls to the 9-1-1 emergency response system [2]. The majority of the calls (68 %) were for medical assistance, while only 4 % of calls were fire-related. In comparison, in 2003, 61 % of calls were for medical assistance and 7 % of calls were fire-related [3]. This distribution of fire service responses reflects broader national trends, which include a decreasing incidence of fires and fire-related injuries [4], along with an increased public reliance on calling 9-1-1 as a point-of-entry into the health care system [1, 5, 6]. The fire service is therefore engaged in activities far beyond fire prevention and control, performing social and medical functions that are key to supporting the public’s health.

Across the U.S., there are 27,140 registered fire departments, which employ 1,065,600 firefighters [7]. They work in a range of organizational structures, with some departments engaging career, paid per call, and/or volunteer workforces [7]. The system is decentralized, with training, protocols, organizational climate, and social context varying tremendously across departments [1]. Departments also structure and organize the relationship between fire and emergency medical service providers—which have various levels of training—in multiple ways. While there is recommended testing for Emergency Medical Technicians (EMTs) and paramedics, along with proficiency standards, through the National Registry for EMTs, there is no industry standard for the number, types, and training levels of EMS staff required within any fire department or community [1]. Within such a decentralized and varied system, fire departments share several characteristics, broadly defined: the work is strenuous, high risk, and provided as a public service. Beyond that, the fire service has become an important out-of-hospital source of emergency medical care. Indeed, nearly half of the nation’s EMS systems are based in fire departments [1].

This work was motivated by theories of safety culture and safety climate, which we employed as frames for studying occupational health and wellbeing within the fire service [8]. Safety culture includes the core norms and values of an organization (e.g., a fire department) with regard to injury control and prevention, as well as related practices that may be ritualized and codified in policies and procedures. Safety culture is a multi-dimensional construct that is shared by members of a group or organization and is relatively stable over time, though it may be amenable to change. Safety climate captures the perceptions that employees share regarding safety-related behaviors, habits, practices, and policies within their work environments. Within a broader parent project on occupational safety climate in the U.S. fire service, we observed that firefighters were consistently and markedly concerned about both the volume and nature of 9-1-1 calls, and about related implications for occupational health, public safety, and community welfare. The goal of this current analysis was to assess firefighters’ public health-relevant concerns regarding the daily demands, challenges, and vulnerabilities of the 9-1-1 emergency response system.

## Methods

### Study Design

We conducted focus groups and interviews with a diverse sample of firefighters from 12 fire departments across the U.S. in order to understand firefighters’ perceptions of job demands, organizational climate, and occupational safety and risk. The current analysis focuses on the public health implications of firefighters’ concerns regarding the 9-1-1 emergency response system, which were consistently raised across all participating fire departments.

### Population and Setting

To achieve a diverse sample, we sought input from an advisory board that included leaders in the fire service from across the country. We recruited departments that varied according to: (1) geographic region; (2) urban, suburban, or rural context, and (3) workforce characteristics (i.e., career, volunteer, or combination workforces). Eighteen departments were invited to participate, and 12 departments joined the study. Within participating departments, leaders identified members (age 18 or over) who were currently in active service and were available for focus group discussions and interviews. Throughout this paper, we refer to participants as “firefighters,” which is often the role with which they most closely identified, even though this group also included emergency medical service providers of all levels—from basic life support EMTs to advanced life support paramedics.

### Human Subject Committee Review

Written informed consent was obtained from all participants. The Drexel University Institutional Review Board and the Department of Homeland Security’s Regulatory Compliance Office approved the study protocol.

### Measurements and Sample Size Determination

Using a semi-structured interview guide that was adapted for career versus volunteer and frontline firefighters versus supervisors, we conducted 10 focus groups and 62 interviews, with a total of 123 participants from the Eastern (n = 59), Central (n = 33), and Western (n = 31) regions of the U.S. Questions addressed perceptions of job risk, departmental safety policies, procedures, outcomes, and attitudes and beliefs regarding occupational safety. During the first phase of data collection, we conducted focus groups with frontline firefighters and one-on-one interviews with supervisors holding the rank of company officer or above. After collecting and reviewing data from seven focus groups, we had reached thematic saturation in focus group data, though one-on-one interviews continued to elicit new information. Despite having reached saturation, we proceeded with three additional previously-scheduled focus groups. We then revised the data collection strategy for all remaining fire departments, to conduct only one-on-one interviews with both frontline firefighters and their supervisors.

### Analytical Methods

Focus groups and interviews were audio-recorded, transcribed, de-identified, and entered into QSR NVivo 10 for analysis [9]. Members of the study team (ADK, ALD, CCC, JAT, YK) developed a codebook based on the interview guide and close reading of five early transcripts. Two trained research assistants (ADK, YK) coded 25 % of the transcripts to assess inter-rater agreement, which exceeded 90 %. Given this high level of agreement, remaining transcripts were coded by a single team member.

The current analysis is based on transcript content coded to two categories: (1) the changing nature of work in the fire service; and (2) the 9-1-1 emergency response system. Using an iterative approach [10–13], three team members (JAT, ALD, CCC) reviewed the data and summarized key observations in memos that were extensively discussed at a series of team meetings. The team reached consensus on data patterns and cross-cutting themes, which are reported in the results: the changing nature of work in the fire service; community expectations regarding the fire service and 9-1-1 emergency response system; practical implications of the 9-1-1 system’s broad social mandate; and emotional/psychological implications for firefighters on the changing nature of their work.

## Results

### Participant Characteristics

Focus group participants (n = 61) were all frontline firefighters and, on average, were 38 years old and had worked as firefighters for 11 years. The majority of interview participants (71 %) held supervisory roles. They were 48 years old, on average, and had worked as firefighters for a mean of 24 years. Ninety percent of the interview participants and 69 % of the focus group participants were men (Table 1).

**Table 1:** Participant and fire department characteristics

Characteristic	% (n)
Participants (interviews and focus groups, n = 123)	
*Sex*	
Male	80 % (98)
Female	20 % (25)
Age (mean/SD)	42.86 ± 10.55 years
*Race/ethnicity*	
Caucasian	83 % (102)
African American	10 % (12)
Asian	0 % (0)
Hispanic	2 % (3)
Other	3 % (4)
No response	2 % (2)
*Education*	
High school	10 % (12)
Some college	19 % (23)
Technical school	6 % (7)
2-year college/associate degree	22 % (27)
4-year college	34 % (42)
Graduate school or more	10 % (12)
*Rank/role*	
Frontline firefighter	64 % (79)
Supervisor	36 % (44)
Fire department (n = 12)	
*Geographic region*	
East	50 % (6)
Central	25 % (3)
West	25 % (3)
*Workforce*	
Career	67 % (8)
Volunteer	17 % (2)
Combination	17 % (2)

### The Changing Nature of Work in the Fire Service

According to the firefighters who participated in this study (“firefighters”), 70–90 % of their departments’ 9-1-1 calls were for EMS runs rather than for fires. One of the study’s most resounding themes is that the work of the fire service has changed radically in recent years. Respondents reported that medical calls have become more common while structure fires have become less common, more quickly contained, and often less severe. In the results below, we draw from firefighters’ commentaries to examine the public health implications of this major shift in the functions of the fire service and the 9-1-1 emergency response system. In Table 2, we refer to research questions raised by firefighters’ commentaries.

**Table 2:** Key themes, representative quotes from firefighters, and research questions derived from firefighters’ commentary on the 9-1-1 system

Key theme	Relevant quote from firefighters	Research questions
*The changing nature of work in the fire service*		
Within the fire service, there is an increased emphasis on emergency medical services (EMS) and a decreased emphasis on firefighting, in part because of the success of the fire service in primary and secondary prevention of fires and fire-related injury.	“Most of our work is in the area of emergency medical services. It reflects a trending change over several decades and now most of the work that we do is emergency medical services. It also reflects what we’ve done in fire prevention over several decades where we work to make sure that the citizens are safe…” “We respond to all types of incidents, everything from car accidents to stuck elevators to the baby stuck in the car, things like that and then on the EMS side, everything from auntie fell down in the kitchen and she needs service, to–uncle’s upset and we don’t know what’s wrong with him and he has a mental illness. So we respond to … [a] full range of humans having crisis. Sometimes, again, it’s the cat up the tree. We still respond to the cat up the tree.”	What is the distribution of 9-1-1 calls for fire, medical, and social assistance? What is the distribution of calls by level of acuity?
As fires have become less common, the fire service has had to adapt, taking on new community service roles.	" If you’re a firefighter who’s only interested in going to fires, for most of us that’s three or four percent of our business nowadays. We’re quickly going to go extinct as an organization if we don’t find more ways to provide service to the community."	Are the resources of the fire service matched to the needs of callers? How should the resources of the fire service be deployed for optimal public health benefit?
*Community expectations regarding the fire service and the 9*-*1*-*1 emergency response system*		
The 9-1-1 system serves as a point of access to primary care for underserved populations.	“We’re seeing a lot of people who don’t have primary care physicians who use us as their primary care physician. I don’t know if they’re consciously doing it or not, but then the ER, by default, becomes the primary care physician’s office instead of an emergent office. It’s become routine medicine at the emergency room and we’re the ambulance company that gets them there.”	What alternatives exist to help citizens access primary care more efficiently, without overuse of the 9-1-1 system or emergency departments? Is the current model the optimal one, in which the 9-1-1 system provides for wide-ranging fire, medical, and social needs in the community? Will changes from the Affordable Care Act shift utilization away from 9-1-1 as a primary care access point?
The 9-1-1 system provides a system of last resort, compensating for a frayed social safety net.	“There’s probably other blame that we can place, I think, on our public aid system. I know there’s abuse where people may have to be transported to an ER facility to get payment as opposed to going to the doctor, which would cost them money perhaps.”	Considering the 9-1-1 system and other publicly-funded medical and social programs, what is the most efficient and cost-effective model for attending to citizens’ fire, medical, and social needs?
Firefighters often assist the most marginalized citizens, who face many chronic challenges and have many medical and social needs.	“I have the homeless shelter in my district, and we go there five times a day, especially in the morning, when somebody needs to go to the hospital and they don’t want to walk. Or in the evening, when they shut the doors and they can’t get back in there. EMS knows the people, they call them every day. Why not send the police instead of sending me.”	What strategies can best improve the health and wellbeing of highly vulnerable (e.g., homeless, poor, or mentally ill) citizens, so that the 9-1-1 system is not necessary as a system of last resort? How can the needs of vulnerable citizens be met while reserving the resources of the 9-1-1 fire/EMS system for emergency response?
Vulnerable elderly citizens are among the frequent callers to the 9-1-1 system, because they often live alone and lack informal or formal sources of support.	“We have elderly apartment complexes around that they call us out all the time just because they need help doing—moving from their wheelchair to the bed, things like that. I mean—and it gets abused and then we have to say, hey, we have to call our administration to say listen. We’ve been out here three times today doing this. They need to be moved to an assisted living facility.”	What systems and structures, in addition to the 9-1-1 system, can best support the medical and social needs of an aging population? How should fire and EMS services be structured, staffed, and trained to meet the needs of an aging population?
*Practical implications of the 9-1-1 system’s broad social mandate*		
Firefighters are concerned that low-acuity calls are common and divert resources that may be needed to attend to higher-acuity emergencies. They believe that tiered or triaged dispatch systems are necessary to match the resources deployed (number and type of personnel, type of equipment) and response times to callers’ needs.	“[We] have to stay with a patient until [an] equal or higher standard of care is given, regardless of whether the call is very serious or not. And we go on some very very not high—very low priority calls… [We] could be on one of these calls…[and] something of much more greater priority and danger, i.e., a fire, a shooting, something, could be right across the street, around the corner, whatnot, and we’re not allowed to leave and go take care of that situation. And it’s been brought to people’s attention in the higher-ups…I don’t know if it fell on deaf ears or on—in hands that are tied. I don’t know.”	What proportion of fire departments have tiered or triaged dispatch systems, assigning level of response to each call? What are the optimal systems and policies for deploying fire service resources where they are most needed?
Firefighters reported that the risks they take—racing to respond to 9-1-1 calls—are often not warranted, given the low acuity of many callers’ needs.	“…It’s dangerous to put a truck in the street on a [low-acuity] response for a headache. It’s equally dangerous to keep firemen up all night and then expect them to respond to a fire, where they actually need to save somebody’s life.”	What proportion of calls to 9-1-1 could be classified as medically or otherwise unnecessary? How do the daily demands of work influence firefighters’ readiness to respond to emergencies?
After many low-acuity 9-1-1 calls, firefighters may become desensitized, responding more slowly to calls or responding unfavorably to citizens’ requests for aid.	“So I just turned on red lights, ran red lights, stopped traffic, for somebody’s knuckles hurting. It’s dangerous, for one, it desensitizes us, we’re going on all these calls and now we—it can really, it’s just human nature, when you do something over and over and over again, you’re expecting it to be a bunch of B.S. When it actually comes for a real call, you know, you might—it can affect you, I’m not saying it does, but it could. I think that’s sort of a dangerous thing that is being instilled in the fire service.”	How do response times vary across departments? How are response times affected by volume of low-acuity calls?
One of the challenges of work in the fire service is that it can shift rapidly from mundane to life-threatening without warning.	“I mean, we run a lot of psych patients, shootings, stabbings, unknown medical alarms, which you never know what you’re walking into then…When these guys go through their EMT class, you’re trained to walk in there and say “scene safe,” but it’s a check mark in the box. So they’re walking in automatically and just say “scene safe” without truly evaluating a scene. Because how often in your career are you actually going to run into an unsafe medical scene? Very few. So I think we’re automatically training people to assume it’s safe, and not actually evaluating.”	What are the particular hazards of non-fire-related work in the fire service? What are the EMS risks, beyond the occupational hazards typically associated with health care work (e.g., beyond needle sticks)? What is the incidence of assaults to firefighters/EMTs/Paramedics?
*Emotional and psychological responses to the daily demands of work in the fire service*		
Morale, and possibly mental health, suffers among firefighters when their work includes a heavy burden of low-acuity 9-1-1 calls.	“Well, yes, you took the job to protect life and property. That’s what you took the job for, and I have no problem helping a person that’s having a heart attack. That’s your job. But to find somebody’s remote that’s an invalid, that’s in my opinion not our job…That’s definitely had an effect on morale.”	What are the mental health risks of work in the fire service? How does morale influence the effectiveness (and occupational safety practices) of firefighters? What are the most effective strategies to maintain the mental health and functioning of firefighters?
Firefighters may use negative coping strategies to handle the stresses they encounter in their jobs.	“I think at some level we need to understand that if we’re going to hire people to run—with the expectation they run into burning buildings and they’re going to walk into people’s homes, and they’re going to hand us their babies and expect us to take care of them, they’re going to do some crazy shit on their days off, and they’re probably going to do some crazy shit while they’re working. It’s hard for them to turn that off.”	What is the incidence of negative coping (e.g., alcohol and drug abuse, depression, anxiety, PTSD) among firefighters? What are the most effective strategies for mitigating the potential adverse mental health consequences of work in the fire service?
Firefighters get frustrated when they routinely run low-acuity calls, because they want to keep their skills sharp, and they want to use those skills where they are most needed.	“Every one of us wants to run calls and everybody wants to help people. We want to do our job. But we want to do something that we’re needed.”	How has the decreasing incidence of fires influenced the skills and job readiness of firefighters? What additional skills are required for today’s EMS providers? What additional skills do firefighters need in order to be better prepared to manage citizens’ medical and social needs?

### Community Expectations Regarding the Fire Service and the 9-1-1 Emergency Response System

The 9-1-1 system serves as a broad health and social safety net, according to study participants—one of whom characterized the fire service as providing “socialized medicine in the community.” Other firefighters explained that the fire service responds to every possible type of community stress, including medical calls of all acuity levels, hazardous materials incidents, extreme weather events, and both literal and figurative cats stuck in trees. A central challenge, according to firefighters, is that there are essentially two systems available to address all medical, social, and safety concerns in the community: police and fire/EMS. Many firefighters accepted that their service was meeting a fundamental, and otherwise unmet, need for care in the community. As one participant explained, calling 9-1-1 is often the community’s best available option for getting help. But as a result, the public uses the 9-1-1 system liberally, not just during emergencies, and firefighters spend substantial time and resources—even the majority of their work time—responding to non-emergent 9-1-1 calls.

Many firefighters recognized that community residents rely on emergency departments for primary care and on the 9-1-1 system for “free” ambulance transport to the hospital. Firefighters often acknowledged that poverty was a root cause of the community challenges they respond to daily. One firefighter described a composite case of a low-income, single mother with a sick child. The firefighter explained, “It’s 2:00 in the morning. And you’re getting called to take this mom and the kid to the hospital…That woman’s making a rational choice. She calls 9-1-1. She gets door-to-door service. She gets first in line in the emergency room. She’s probably sent home with the medication she needs for the kid and she’s given a cab voucher back. That’s one smart woman. Otherwise what are her other options?” The firefighter understood that poverty and a flawed social safety net led to the mother’s reliance on the 9-1-1 system for non-acute care, and that this type of reliance on the 9-1-1 system diverted fire service resources and personnel from response to higher-acuity emergencies.

Firefighters also noted that the aging of the population is a major contributor to high 9-1-1 call volumes. Calls often come from elderly citizens who live alone and lack both family support and the financial resources to secure in-home assistance. Both poverty and an aging population were viewed as drivers of the “frequent flyer” phenomenon, wherein certain vulnerable citizens repeatedly call 9-1-1 for assistance for non-emergent conditions or even for assistance with activities of daily living. Firefighters were critical of the lack of both informal (i.e., family, friends) and formal structures to provide daily support for elderly and infirm community residents, noting that even assisted living facilities rely on the 9-1-1 system rather than having their own personnel respond to nonserious resident issues (like falls without apparent injury)—driven in part by legal liability concerns rather than resident needs.

### Practical Implications of the 9-1-1 System’s Broad Social Mandate

Many firefighters expressed concern and frustration that responding to non-emergent 9-1-1 calls diverts resources from emergency response. While many departments proudly embraced an “all hazards” response and expected their members to respond with alacrity to any community need, firefighters routinely reported that they wanted to be deployed where their specialized skills were most needed. They were frustrated when their 9-1-1 dispatch systems and protocols did not allow for a tiered or triaged system of response that could assign non-emergent calls a lower priority and slower response time than high-acuity medical calls, fires, or traumas.

Firefighters reported a sense of duty to respond quickly to every call, as if it were an emergency. In most focus groups and interviews, firefighters commented on the hazards of speeding through traffic “running lights and sirens.” According to firefighters, these risks were unwarranted for low-acuity calls that they likened to “literally, a hang nail” or “my back hurts.” Firefighters identified an important gap in departmental policies—because controls are often not in place to triage calls and convey the level of acuity (and the corresponding need for speed) to the responding firefighters.

In addition, some firefighters reported being “desensitized” by non-emergent 9-1-1 calls, especially those originating from familiar, often-visited addresses. As one firefighter said, “I have the homeless shelter in my district, and we go there five times a day, especially…when somebody needs to go to the hospital and they don’t want to walk. [Or] when they shut the doors and they can’t get back in [the shelter]…It’s just a revolving door there and it always seems to be an emergency where I have to turn the lights on, or I have to run red lights and stuff. Then you just get…desensitized, I guess. It’s not one of my favorite places to go anymore.” Another firefighter suggested that he resented and only minimally engaged with patients on those repeat or routine calls, explaining that “I’m just going to stare at you and I’m going to look around your house and we’re not going to talk. It’s the most awkward ride to the hospital you’ll ever get”—suggesting the potential for lower-quality care and treatment of marginalized or vulnerable patients who become identified as high utilizers of the 9-1-1 system.

One of the core challenges of work in the fire service is that a day’s work can suddenly shift from tedious—including “routine” or “BS” runs—to extremely dangerous calls, even in the absence of fire emergencies. Firefighters reported that the risks to their personal safety can escalate rapidly, especially when they encounter “psych patients, shootings, stabbings, unknown medical alarms.” As one firefighter said, “you never know what you’re walking into…[and] you’re trained to walk in there and say ‘scene safe,’ but it’s a check mark in the box…without truly evaluating a scene.” This is in contrast to firefighters’ keen situational awareness at the site of fires, for which they have been trained to comprehensively evaluate the particular risks of each new event. Social and medical emergencies, especially those involving drugs, alcohol, guns, and violence, posed dramatic and highly unpredictable occupational hazards for firefighters, and they often felt exposed to those risks, especially if their departmental protocols did not also call for police accompaniment to the scene.

### Emotional and Psychological Responses to the Daily Demands of Work in the Fire Service

The fire service is a high-demand, high-stress work environment. Morale suffered among firefighters who perceived that the public misused the 9-1-1 system. One firefighter estimated that “probably 90 percent of their calls are not true emergencies,” adding that callers sometimes use the “magic word” claiming to have chest pain to get immediate attention, even if “it’s really their knee that hurts.” As another firefighter said, “what we cannot tolerate and what is destroying the morale of this company and other fire companies is that…we are waiting 25 min for an ambulance to come and take someone who should have been going to a medical facility in a taxi cab”.

Nonetheless, many firefighters, driven by a sense of mission and responsibility to the community, accepted the full range of roles inherent in their work. In both firefighting and responding to EMS calls, participants reported the highest degree of satisfaction when they were able to use their specialized skills to respond to high-acuity emergencies. Firefighters explained that, in part, they like “the adrenaline rush, as opposed to it being mundane.” In addition, they want to practice and improve their technical firefighting skills, and they do not want those skills to be wasted or to atrophy from disuse. And as one firefighter noted, “interestingly enough…you won’t hear them complain about a big traffic crash with four cars involved…you don’t hear them complain about those EMS calls…you don’t hear them complain about the CPR calls, or shooting, or stabbings,” because those “exciting” calls reflect firefighters’ expectations regarding the kinds of highly skilled work they expect their jobs to entail. Firefighters did, however, consistently express concern about being unavailable to respond to high-acuity calls when they were engaged in responding to lower-severity community needs—as if they were missing their opportunity to do the work they signed on to do, while attending to tasks and community needs that were not necessarily included in their job expectations or their employment training.

## Discussion

In our conversations with firefighters for a study about occupational safety climate in the fire service, a resounding theme emerged. Firefighters across the country reported concerns about the changing nature of their work, especially with regard to the 9-1-1 emergency response system. Firefighters consistently described the 9-1-1 system as a broad social safety net that responds to high-volume calls to address both fires and the community’s varied medical and social needs. A consistent portrait emerged of a system strained by the otherwise unmet needs of our community residents, especially those who are elderly and poor, for whom calling 9-1-1 is often the only path to obtaining much-needed immediate assistance. Firefighters described a broken 9-1-1 system, in which the community’s needs and expectations often do not match the skills, training, expectations, and resources of today’s fire service—which in turn affects the degree to which the fire service is able to support both the community’s health and the occupational health of firefighters. The work reported here focuses on the daily, routine demands faced by the fire service, but these findings clearly have important further public health implications regarding the readiness of our 9-1-1 system to respond to extreme or mass casualty events. As the first public health inquiry to report on firefighters’ in-depth reflections on our current 9-1-1 emergency response system and its challenges, this work raises several major issues for public health.

First, these findings raise the central question of what we, as a society, want and expect from the fire/EMS 9-1-1 system. Do we want the 9-1-1 system to continue to serve as an “all-hazards” response system—which includes responding to catastrophic events as well as serving as a point of primary care and social support in the community? Firefighters in this study were highly critical of this model in its current form—especially because existing protocols and policies in many departments treat every 9-1-1 call as an emergency, when many calls clearly are not highly time-sensitive emergencies. Several states have enacted laws to discourage calling 9-1-1 for non-emergent reasons [14], though the effectiveness of these laws and the extent to which they are enforced is unclear, and no study participants referred to these laws. Systems of triage, tiered dispatch, and deployment of appropriate personnel and equipment are necessary so that the fire/EMS response can be tailored to meet callers’ needs while optimizing use of resources and maintaining emergency readiness. Emerging models of “community paramedicine” include strategies for managing citizens’ medical and social needs proactively with pre-planned, non-emergent responses by specially trained paramedics and EMTs, thereby preempting potential medical crises and decreasing utilization of the 9-1-1 system, and subsequently the emergency department, for non-emergent issues [15, 16]. Solutions to the overuse of in-hospital emergency medical care must be crafted in close consideration of prehospital emergency care delivered through the 9-1-1 system. If, as a society, we instead want a more circumscribed role for firefighters and EMS—if we want them only to respond to true emergencies—then we have a major task to undertake in reshaping social norms regarding 9-1-1 calling patterns and habits.

A second issue raised by this paper is that firefighters face specific EMS-related occupational risks, many of which are engendered or exacerbated by the high volume and tremendous variability of calls to the 9-1-1 system. Injury rates are high due to many threats, including sprains and strains, exposure to infectious bodily fluids, and assault by patients or bystanders [1]. Moreover, driving to an emergency in itself carries risk and represents the leading cause of occupational fatalities among firefighters [17]. Fatal and injurious crashes involving firefighters and EMS personnel are an important public health concern—one that was frequently voiced by study participants [1, 18–20]. Much is demanded of firefighters, EMTs and paramedics, and it is incumbent upon public health professionals to identify the best ways to respond to and mitigate the occupational hazards and stresses of the job. Situational awareness is second-nature to seasoned firefighters who are trained to foresee potential hazards at the scene of a fire, and protocols are in place to prevent over-exertion. In contrast, there are few broadly deployed programs to help firefighters deal with the psychological and emotional impacts of their work or to mitigate the unnecessary risks of “lights and siren” responses to non-emergent calls.

Third, firefighters’ training should better match the demands they currently face on the job. According to paramedic M. Womble, MHA (written communication, June 2015), with the exception of the National Registry for EMTs, which offers a widely accepted standard of education and competency, there are no accreditation requirements for EMS training programs and there is no nationally accepted standard regarding how EMS should be established, delivered, and maintained across the nation’s diverse communities [1]. Typically, firefighter training, and associated budgetary expenditure, is focused principally on fire suppression [1], with specialization in several areas (e.g., extrication, hazardous materials, and swift-water and high-angle rescue). EMS has its own specialized demands and calls for technical expertise in determining treatment pathways as well as in navigation of the complex social or interpersonal issues commonly faced. Given the range of daily challenges firefighters report, there is a need for public health and social work expertise within the fire service [21]. Therefore, the development of the following skill sets and policies would support the now-routinely demanded sociomedical work of the fire service: community-level needs assessment; systems for referral of citizens to appropriate health and social services; evaluation of the safety and condition of housing; and identification of immediate social threats in the community (e.g., domestic violence). The fire service has been remarkably successful in advancing the primary and secondary prevention of fires and fire-related morbidity and mortality [2, 4]. That prevention orientation should transfer into EMS work. Given that EMTs are privy to citizens’ private lives and living conditions in ways that other health care providers are not, they are well-positioned to have an impact on identifying risks and improving the conditions for health in homes and communities.

This study, as a qualitative analysis, raises important challenges for the country’s 9-1-1 system but cannot answer critical questions regarding evidence-based strategies to improve the delivery of both high- and low-acuity medical and social care in the community. Additionally, firefighters from career versus volunteer departments, or from different regions, or from departments with and without tiered dispatch systems and triage, have not been explicitly compared (for patient outcomes) to evaluate the efficacy and cost-effectiveness of those alternative delivery models. Finally, while our sample is large for a qualitative study, and while it is diverse in many ways, it is possible that we have omitted important perspectives, such as the views of industry leaders who have developed and implemented progressive 9-1-1 policies and practices that are not adequately reflected here.

## Conclusion

This paper raises the critical need for our society to address the root causes of a dependence on the 9-1-1 system to attend to non-emergent medical needs. This is especially important because the challenges described in this paper are routine, daily challenges that present potential threats to both community and occupational health—and leave the impression that our system’s “surge capacity” to respond to acute and/or large-scale emergencies may be taxed. As firefighters in this and other studies have noted, the challenges of our country’s poor and elderly citizens are not met by other existing safety nets [5]. The importance of strengthening the lives and livelihoods of our vulnerable populations—and increasing their access to needed opportunities and resources—is indeed an undercurrent in this report. Currently, and often as a stopgap measure, our citizens call 9-1-1 when in need. We would be wise to heed the concerns of the men and women who respond to those calls.
